# A collaborative pharmacist-led intervention to prevent hospital readmissions among elderly patients discharged from the emergency department: a retrospective cohort study

**DOI:** 10.1038/s41598-024-64968-8

**Published:** 2024-07-03

**Authors:** Susan Tran-Nguyen, Stephen Edward Asha

**Affiliations:** 1 Pharmacy Department, St George Hospital South Eastern Sydney Local Health District (SESLHD), Kogarah, Sydney, 2217 Australia; 2 Emergency Department, St George Hospital South Eastern Sydney Local Health District (SESLHD), Kogarah, Sydney, 2217 Australia

**Keywords:** Health services, Medical research, Outcomes research

## Abstract

Unplanned hospital readmission is a safety and quality healthcare measure, conferring significant costs to the healthcare system. Elderly individuals, particularly, are at high risk of readmissions, often due to issues related to medication management. Pharmacists play a pivotal role in addressing medication-related concerns, which can potentially reduce readmissions. This retrospective single-centre cohort study, conducted from November 2022 to February 2023 in an emergency department, aimed to determine if integrating emergency medicine pharmacists into Emergency Department care models reduces unplanned hospital readmissions within 28 days and to identify the interventions they employ. The inclusion criteria included patients aged ≥ 65, taking ≥ 3 medications, and presenting with falls, cognition changes, or reduced mobility and were planned for discharge to home from the emergency department. Collaborating with the Emergency Department Aged Care Service Emergency Team, a pharmacist provided comprehensive medication management consultations, discharge liaison services, and other pharmacy related interventions to eligible participants whenever the pharmacist was available. Patients who met the eligibility criteria but did not receive pharmacist interventions due to the pharmacist's unavailability served as the control group. This method was chosen to ensure that the control group consisted of comparable patients who only differed in terms of receiving the pharmacist intervention. The study included 210 participants, with 120 receiving pharmacist interventions and 90 acting as controls. The results revealed a significant reduction in unplanned hospital readmissions among participants who received pharmacist interventions (10.0%, n = 12) compared to controls (22.2%, n = 20), with a notable difference of 12.2% (95% confidence interval 2.4–23.4%, *p* = 0.01). A total of 107 interventions were documented, emphasising medication selection recommendations (28.0%) and identification of adverse drug reactions/drug-drug interactions (21.5%) as primary areas of focus. These findings suggest that integrating skilled pharmacists into Emergency Department Aged Care Service Emergency Team (ASET) lowered the rate of unplanned hospital readmission within 28 days resulting in improved hospital performance metric outcomes. This highlights the potential role of pharmacists in addressing medication-related issues and enhancing the quality and safety of healthcare delivery, particularly for elderly patients transitioning from the ED to home care settings.

## Introduction

In Australia, the rates at which patients are readmitted to the same facility within 28 days of discharge are a key performance indicator for safety and quality in healthcare^1,2^. Similar approaches for quantifying the performance of public health systems have been adopted in England, Scotland, the US and Canada^3,4^. Data from America^5^, France^6^, Belgium^7^, and Taiwan^8^ have reported readmission rates at 28 days to 6 weeks ranging from 10.7 to 23.6%. In 2015, the unplanned 28-day readmission rate from state health systems in Australia ranged from 3.2^9^ to 10.9%^2,3,10^. Unplanned readmissions have a substantial financial burden on the healthcare system,^3,4^, estimated to cost Australia in the order of $1.5 billion annually^11^. Unplanned readmissions are also associated with overcrowding, hospitalisation-acquired complications and infections, iatrogenic harms, and poor health outcomes^2,12^.

Older patients are a high-risk group for hospital readmission^13^. The Australian Institute of Health and Welfare has estimated people aged 65 years and over accounted for 46% of all potentially preventable hospitalisations, and medication management was identified as a risk factor^14^.

Pharmacists are involved in medication history taking, medication reconciliation, and are in an optimal position to identify and address medication-related problems. They can identify and provide advice to manage polypharmacy, drug side-effects, drug interactions, identify deprescribing opportunities, and engage patients in medication education to improve knowledge and compliance, interventions that have been shown to reduce readmissions^15,16^.

In 2022, the Deeble Institute for Health Policy Research Queensland published a comprehensive review on strategies to mitigate unplanned hospital readmissions. The review concluded a model of care that employed multidisciplinary teams and multifaceted approaches resulted in improved patient care and were more successful in reducing unplanned readmissions^12^. Moreover, the tangible benefits of this approach have been exemplified in studies involving specific disease states, showcasing the efficacy of a collaborative strategy involving pharmacists in mitigating readmissions^17–19^.

In light of these insights, the concept of integrating emergency medicine pharmacists into allied healthcare teams within the Emergency Department (ED) emerges as a promising solution for reducing unplanned readmissions.

The primary aim of this study was to determine whether integrating an emergency medicine pharmacist into existing ED care teams that manage older patients led to a reduction in unplanned hospital readmissions within 28 days. A secondary aim was to examine the types of interventions carried out by the ED pharmacist.

## Method

### Study design

This study involved collecting prospective data collected from the quality improvement initiative and then reviewing patients' electronic medical records 28 days later to assess if they had any readmissions. It was conducted at the St George Hospital ED in Sydney, Australia. The quality improvement initiative occurred over a four-month period from November 2022 to February 2023. The project plan was reviewed by the South Eastern Sydney Local Health District Human Research Ethics Committee, and advised that as this was a quality improvement initiative it was exempt from formal ethics committee review. Permission to conduct an interview with participants by the pharmacist was sought as part of the clinical practice interaction. Written informed consent was deemed unnecessary by the ethics committee. This study was carried out in accordance with the standards for quality improvement reporting excellence (SQUIRE 2.0)^20^.

### Participants

Patients were eligible for the study if they were referred to the Aged Care Service Emergency Team and/or the quick response program and were taking at least three chronic disease medications. Aged Care Service Emergency Team is an allied health service that supports elderly patients transitioning back to their own homes after discharge from the ED. The Aged Care Services Emergency Team works collaboratively with ED staff, participating in patient assessment and treatment. Quick response program, is an extension of Aged Care Service Emergency Team, focuses on providing short-term services to promote safe and independent living at home, aiming to reduce readmissions. Quick response program ensures that any pharmacy recommendations made during the ED presentation are implemented at home. Referral criteria are aged 65 years or older who are in the ED, are being considered for discharge home, and have one or more of the following: falls within the past 12 months, changes in cognition or behaviour, or concerns regarding safe mobility. Patients admitted to the hospital or discharged to nursing homes were not assessed by Aged Care Service Emergency Team and were excluded from the study.

### Study group allocation

Participants were assigned to either the intervention or control groups based on the order of their referral, following a first-referred, first-intervened basis. Allocation decisions were made on each study day, with assignments to the intervention and control groups determined by the pharmacist’s availability. This approach aimed to minimize potential biases by ensuring that external factors, such as changes in the emergency department's patient load or staffing levels, did not influence the assignment process.

Each day, as patients were referred to the study, decisions were promptly made regarding their inclusion in the intervention group. Patients who did not receive the intervention on the same day were placed in the control group. By making these allocation decisions simultaneously, we aimed to balance the groups with respect to time-related variables and external conditions, thus enhancing their comparability.

On average, six patients were referred daily. The pharmacist did not have time to see all patients but included an average of four patients daily based on the time of referral. Patients the pharmacist did not have time to see were included in the control group.

### Pharmacy intervention group

Patients who met the above criteria and received the pharmacy intervention based on the pharmacist availability were assigned to the intervention group. The intervention was delivered opportunistically by a single part-time pharmacist when available during business hours to conduct a comprehensive medication management. The pharmacist was available Monday, Tuesday and Wednesday each week. All participants in this group underwent a thorough medication history, sourced from at least two reliable references and medication reconciliation upon admission. Additionally, their attitudes toward medications and health were assessed to ensure sound knowledge of medications and compliance. Moreover, pathology review (renal and hepatic) was conducted for potential dosing adjustments as needed.

Types of pharmacist-led interventions included:

1. *Change medication order/clarify medicine* Instances where adjustments were made to the prescribed medication or inquiries were made to ensure clarity regarding a particular medicine.
2. *Medication selection recommendation* Recommendations put forth by the pharmacist that are clinically appropriate for the patient.
3. *Discharge liaison service* Pharmacy related services provided that facilitates the transition of patients from the hospital back to their homes or alternative care settings.
4. *Therapeutic duplication* Identification and rectification of instances where patients were prescribed multiple medications with similar or same therapeutic class.
5. *Overdose* Instances where the prescribed dosage exceeded the recommended guidelines.
6. *Subtherapeutic dose or duration* Cases where the prescribed dosage or duration of medication was insufficient for optimal therapeutic effect as per recommended guidelines.
7. *Adverse drug reactions or drug–drug interaction* Identification and management of adverse reactions.
8. *Administrative issues* Addressing administrative challenges related to medication dosage forms and providing appropriate dosage form alternatives.
9. *Medication education* Provision of education and information to patients regarding their medications, including dosage, administration, and potential side effects.
10. *Deprescribing* Initiatives aimed at reducing or discontinuing medications that are no longer clinically necessary or may pose risks when used long term.

On discharge, the medication reconciliation review included the following (focusing on new therapy):

- Accuracy and completeness of the clinical provider completed discharge medication reconciliation by comparing home medications with new orders at discharge.
- Review financial barriers of certain medications to ensure compliance after discharge.
- Arrange a supply of discharge medication where needed
- Identify drug interactions of newly commenced medications with home medications
- Counselling patients/caregivers on the purpose and appropriate doses of medications. Provide written treatment plan and written information about the medications.
- If there were changes to medications for a patient who had a dose administration aid, the ED pharmacist would follow-up with the patient’s local pharmacy to ensure that change to medication was communicated.

The quick response program team ensured that patients who were discharged had their pharmacy recommendations implemented.

Pharmacist documentation was a part of the intervention. Upon admission, the ED pharmacist would create a comprehensive medication list, which the ED medical officer would then use to reconcile medications. Following the completion of the medication management process, a detailed summary of the patient, including identified issues, recommendations, medication compliance review, and the updated medication list, would be documented in the patient's electronic medical record, and identifiable by the title 'Pharmacy Progress Note'. This documentation was accessible to all hospital clinical providers.

### Control group

Control participants were patients referred to Aged Care Service Emergency Team on Mondays, Tuesdays and Wednesdays during the pharmacist’s working hours during the study period. However, they were not seen by the pharmacist as they presented at a time when the pharmacist’s services was not available due to competing demands.

### Outcomes

The primary outcome was unplanned readmission to the ED within 28 days. Secondary outcomes included examining the types of interventions carried out by the ED pharmacist.

### Data collection

The Aged Care Service Emergency Team maintained a record of all referrals made to the service as part of routine care. Participants were identified from this list. The ED Pharmacist maintained a record of those who received the intervention, as well as the type of interventions conducted. All other data were collected retrospectively from the electronic medical record, including the date of presentation, sex, age, degree of dependency (lives alone or with support), the reason for initial presentation, number of chronic medications on presentation, number of comorbidities, readmission within 28 days, and reason for readmission.

### Statistical analysis

Descriptive categorical data are presented as total counts and proportions, while continuous variables are nonparametric and are presented as medians with interquartile ranges (IQRs). The primary outcome was presented as a difference in proportions with a 95% confidence interval and compared using the chi-square test. This analysis was performed using Microsoft Excel.

### Ethics approval

The SESLHD Research Ethics Committee has confirmed that no ethical approval is needed.

## Results

**Figure 1:**
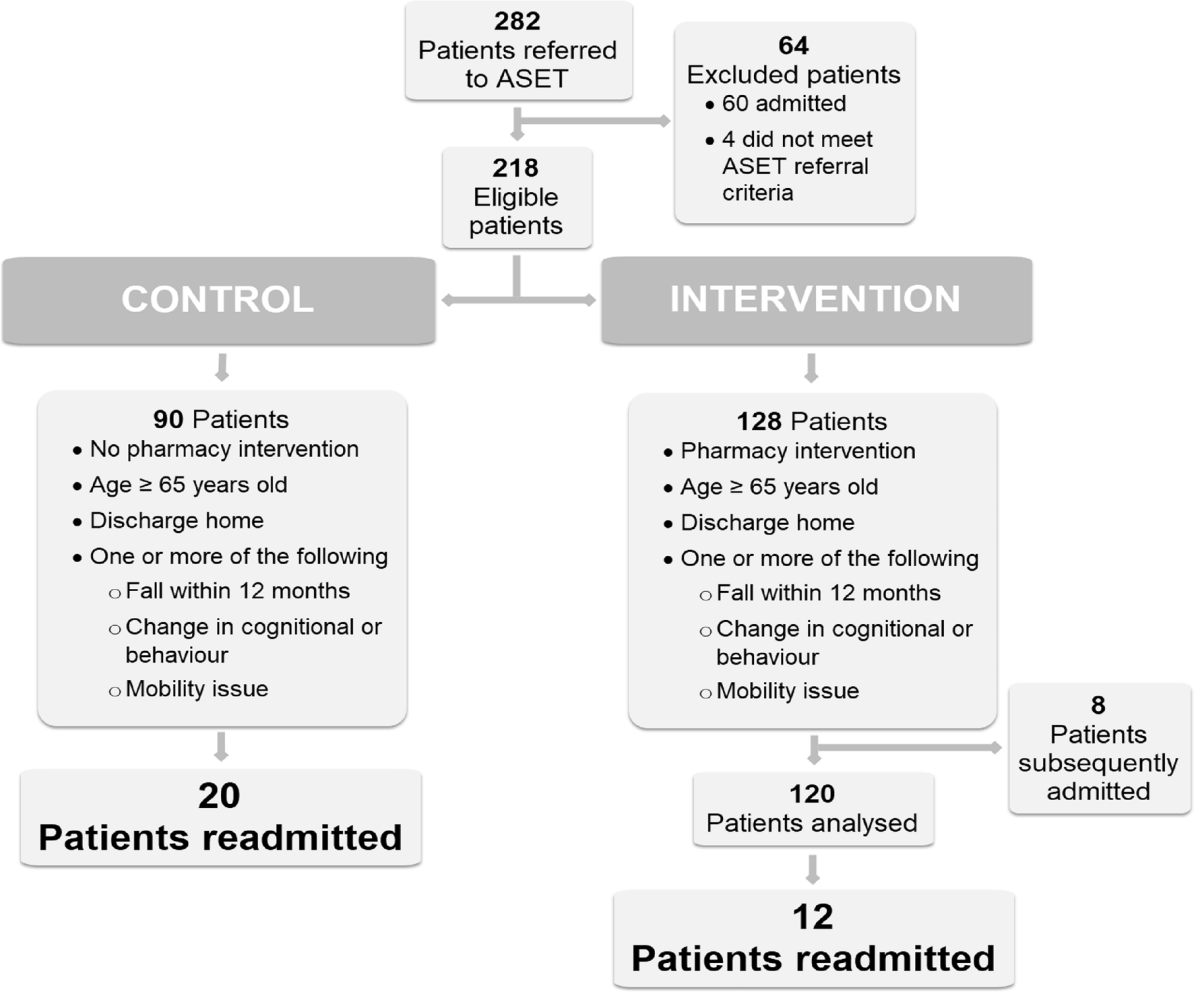
Flow chart.

During the study period, Aged Care Service Emergency Team received 282 referrals, and 218 met the eligibility criteria. Pharmacy interventions were conducted on 128 patients; however, eight were subsequently admitted after review and excluded. This left 120 participants in the intervention group and 90 in the control group, as illustrated in Fig. 1. Table 1 shows the characteristics of the study participants. Unplanned hospital readmission occurred in 12 participants (10.0%) in the intervention group and 20 participants (22.2%) in the control group, a difference of 12.2% (95% confidence interval 2.4–23.4%, *p* = 0.01).

**Table 1:** Characteristics of the study participants.

	Intervention group n = 120	Control group n = 90
	Median (IQR)	Median (IQR)
Age (years)	84 (79–88)	84 (79–88)
Medications on admission	7 (5–11)	8 (4–11)
Comorbidities	5 (3–8)	5 (3–8)

	n (%)	n (%)
Female sex	82 (68.3)	58 (64.4)
Mobility		
Independent	82 (68.3)	64 (71.1)
Assisted	35 (29.1)	23 (25.5)
Dependent	3 (2.5)	3 (3.3)
Living situation		
Alone	68 (56.7)	43 (47.7)
With family	52 (43.3)	47 (52.2)

For those with a primary outcome event, the median time from first presentation to readmission was 14 days (IQR 7–22.5) in the intervention group and 16 days (IQR 8–22) in the control group. Table 2 presents reasons for initial presentation and subsequent readmission.

**Table 2:** Admission reasons (initial ED presentation and ED readmission within 28 days from initial discharge from ED).

	Intervention group n = 120	Control group n = 90
Initial admission reason	n (%)	n (%)
Falls	45 (37.5)	40 (44.4)
Pain	38 (31.6)	27 (30.0)
Reduced function	16 (13.3)	8 (8.9)
Gastrointestinal related causes	7 (5.8)	4 (4.4)
Infection	4 (3.3)	4 (4.4)
Other	10 (8.3)	7 (7.8)

	n = 12	n = 20
Readmission reason	n (%)	n (%)
Fall	2 (16.6)	4 (20.0)
Pain	3 (25)	6 (30.0)
Gastrointestinal related causes	3 (25)	2 (10.0)
Infection	1(8.3)	3 (15.0)
Reduced function	2 (16.6)	2 (10.0)
Other	1 (8.3)	3 (15.0)

The study’s primary outcome, the risk ratio, was calculated to be 0.45 with a 95% confidence interval of 0.233–0.871. This indicates that the risk of unplanned hospital readmission within 28 days is approximately 45% lower in the intervention group compared to the control group. Additionally, the number needed to treat (NNT) to prevent one older adult from being readmitted within 28 days was estimated to be 8.17. This means that for every eight patients receiving the intervention, one fewer readmission would be expected within 28 days.

The secondary aim was to examine the types of clinical interventions made by the pharmacist’s participants in the intervention group. A total of 107 interventions were recorded and are shown in Table 3. Among these, medication selection recommendations were the most common, at 28.0% (n = 30), followed by adverse drug reactions and drug‒drug interaction recognition, at 21.5% (n = 23). Over 93% of the recommendations were proposed and implemented by the ED physician.

**Table 3:** Types of pharmacy intervention.

Intervention types	n (%)
ADRs or drug–drug interaction	23 (21.5%)
Change medication order/clarify medicine	3 (2.8%)
Deprescribing	5 (4.7%)
Discharge liaison service	12 (11.2%)
Medication education	22 (20.5%)
Medication selection recommendation	30 (28.0%)
Subtherapeutic dose or duration	8 (7.5%)
Overdose	1 (0.9%)
Therapeutic duplication	1 (0.9%)
Administrative issues	2 (1.8%)

## Discussion

In this study, we integrated a pharmacist into current Aged Care Service Emergency Team. The pharmacist’s primary focus was on optimising all aspects of patient medication management. While pharmacist interventions are not novel, what distinguishes our study is the strategic placement of a pharmacist within the Aged Care Service Team model and multifaceted pharmaceutical intervention, including medication review and interview. It's important to note that many studies on readmissions tend to focus on patients admitted to the hospital rather than those seen solely in the emergency department. However, the increasing trend of readmissions to the emergency department within one month, rising from 2.4% in 2010 to 3.1% in 2014, is concerning ^21^. Additionally, the elderly cohort represents the highest proportion of these readmission with statistics revealing a 6.2% per annum rise in readmission rates among those over 85 years of age ^21^, emphasising the urgent need for improved streaming processes within the hospital system to prevent these patients from being repeatedly assessed in the ED.

Potential solutions may include implementing multidisciplinary assessment areas, or enhancing geriatric outreach services. Our study contributes to this conversation by demonstrating that proactive interventions, such as integrating pharmacists into the Aged Care Service Emergency Team, can effectively reduce unplanned hospital readmissions. This highlights the potential benefits of such strategies in improving patient care and alleviating burdens on the healthcare system.

We found that the intervention was associated with a reduction in unplanned hospital readmissions within 28 days. This suggests that integrating pharmacists into models for managing older patients can optimise medication management and address potential issues that may lead to readmissions.

Several studies have examined the role of pharmacists within other interdisciplinary and multidisciplinary teams and have demonstrated reductions in hospital readmissions which aligns with our study^17–19,22,23^. These investigations have highlighted a diverse range of interventions orchestrated by pharmacists, who collaboratively engage with medical specialists, consultant nurses, and allied health professionals.

For example, several chronic obstructive pulmonary disease (COPD) specific care bundles that have investigated the inclusion of pharmacists as a strategy to decrease readmissions, with varying degrees of success reported in the literature. In a study by Gentene et al., it was found that pharmacists play a pivotal role in hospital-based transitions of care to lower COPD readmissions ^18^. The integration of a multidisciplinary team incorporating pharmacists into the care plan led to a reduction in COPD hospital 30-day readmission rates, from 22.7 to 14.7%^18^.

Similarly, Thurston et al. found that adding a pharmacist to the transitional care team that managing heart failure patients decreased the readmission rate from 33.7 to 21.3% in the intervention group^17^. The intervention group received the pharmacist-led intervention during their hospital stay and post discharge. The study also found an increase in self-reported patient medication adherence^17^.

Our study found that the Number Needed to Treat (NNT) is 8, meaning that for every 8 patients receiving our intervention, one fewer readmission would be expected. This indicates a potential benefit of our intervention in reducing hospital readmissions within 28 days.

According to the National Hospital Cost Data Collection (NHCDC), the average cost per emergency department presentation in 2020–21 is $789^24^. Therefore, if we effectively reduce readmissions within this cohort, the potential cost savings would be substantial.

Contributions from the pharmacist’s interventions may have influenced patients' initial presentation or, if left unidentified, could have potentially exacerbated their conditions, necessitating further medical attention. This demonstrates that the role of pharmacy practice extends beyond merely compiling medication histories and conducting patient interviews. However, as an integral component in preventing readmissions and highlighting the proactive stance in recognising medication-related issues may avert potential harm that could lead to repeat presentations.

## Limitations

There are several limitations that need to be considered when interpreting the results of this study. This was a relatively small study, with an intervention carried out in a single institution by a single pharmacist, which limits the generalisability to other institutions and pharmacists. The data collected focused solely on readmissions within the hospital where the study was conducted, disregarding occurrences at other hospitals. Consequently, the data might underestimate the actual rates of readmissions.

This retrospective cohort study involved participants selected for the intervention on an opportunistic basis. It is acknowledged that the allocation method of first-referred, first-intervened is not equivalent to random allocation and may introduce selection bias. Specifically, this opportunistic selection process may have resulted in systematic differences between the intervention and control groups. Participants chosen to receive the intervention might have differed in their characteristics, reasons for hospital admission, and potential for having a medication-related reason for presentation or representation. Consequently, observed differences in outcomes may partly reflect these underlying differences rather than the effect of the intervention itself. To address this limitation, we have described the allocation process in detail in the method section. Despite these efforts, it is possible that selection bias influenced our findings, and this should be considered when interpreting the results.

Future studies should aim to use random allocation methods to more robustly assess the impact of pharmacist interventions. Randomised controlled trials would provide stronger evidence by minimising selection bias and ensuring comparable groups.

## Conclusion

In this retrospective cohort study, the inclusion of a pharmacist in the Aged Care Service Emergency Team, responsible for managing older patients being discharged from the ED, was associated with a reduction in unplanned hospital readmissions within 28 days following discharge from the ED. These findings suggest that a collaborative intervention between healthcare professionals may have a positive impact on patient-centred care. To further validate and generalise these findings, a larger randomised multicenter study is necessary.

## Data Availability

The datasets generated during and/or analysed during the current study are available from the corresponding author upon reasonable request.
